# 3D Composite U(VI) Adsorbents Based on Alginate Hydrogels and Oxidized Biochar Obtained from *Luffa cylindrica*

**DOI:** 10.3390/ma16196577

**Published:** 2023-10-06

**Authors:** Andreas Ayiotis, Efthalia Georgiou, Panagiotis S. Ioannou, Ioannis Pashalidis, Theodora Krasia-Christoforou

**Affiliations:** 1 Department of Mechanical and Manufacturing Engineering, University of Cyprus, 1 Panepistimiou Avenue, 2109, Aglantzia, P.O. Box 20537, 1678 Nicosia, Cyprus; ayiotiss@gmail.com (A.A.); ioannou.s.panagiotis@ucy.ac.cy (P.S.I.); 2 Department of Chemistry, University of Cyprus, 1 Panepistimiou Avenue, 2109, Aglantzia, P.O. Box 20537, 1678 Nicosia, Cyprus; georgiou.efthalia@ucy.ac.cy (E.G.); paschalidis.ioannis@ucy.ac.cy (I.P.)

**Keywords:** alginate hydrogels, uranium adsorption, *Luffa cylindrica* fibers, biochar

## Abstract

3D naturally derived composites consisting of calcium alginate hydrogels (CA) and oxidized biochar obtained from *Luffa cylindrica* (ox-LC) were synthesized and further evaluated as adsorbents for the removal of U(VI) from aqueous media. Batch-type experiments were conducted to investigate the effect of various physicochemical parameters on the adsorption performance of materials. The maximum adsorption capacity (q_max_) was 1.7 mol kg^−1^ (404.6 mg·g^−1^) at pH 3.0 for the CA/ox-LC with a 10% wt. ox-LC content. FTIR spectroscopy indicated the formation of inner-sphere complexes between U(VI) and the surface-active moieties existing on both CA and ox-LC, while thermodynamic data revealed that the adsorption process was endothermic and entropy-driven. The experimental data obtained from the adsorption experiments were well-fitted by the Langmuir and Freundlich models. Overall, the produced composites exhibited enhanced adsorption efficiency against U(VI), demonstrating their potential use as effective adsorbents for the recovery of uranium ions from industrial effluents and seawater.

## 1. Introduction

Uranium is a naturally occurring, primordial heavy metal ion that can be found in various ratios in rocks, soil, and water. Its toxicity and radioactivity are major concerns for public health, and its extensive usage in the nuclear fuel cycle results in the contamination of wastewater and the surrounding environment [1,2].

Different methods have been applied for the removal of (radio)toxic metal ions from aqueous media, including adsorption, ion exchange, electrodialysis, membrane filtration, etc. [3,4,5,6,7]. Among those, adsorption—which has been extensively used in the effective removal and recovery of U(VI) from the ecosystem [8,9,10,11,12,13,14]—is the most efficient, undemanding, cost-effective, and environmentally friendly process [15]. The efficiency of this method is highly dependent on the type of adsorbent used with respect to its chemical composition and morphology [16]. Examples include naturally derived fibers [17,18,19], electrospun fibers [20,21,22,23], porous organic polymers [24], and hydrogels [25]. The latter has attracted considerable attention in the last years as effective adsorbents for the removal of harmful metal ions from aquatic environments [26,27,28], due to their highly hydrated 3D structure and tunable chemical composition that enable the incorporation of specific metal binding moieties such as carboxylic acid [29,30], hydroxyl [31], amidoxime [32,33,34], vinyl pyridine [35], β-ketoester [36], and amino functionalities [37,38,39], exhibiting high affinity for various metal ions of different geometries and oxidation states. Besides the development of chemically crosslinked hydrogels destined for use in metal ion removal, eco-friendly, physically crosslinked hydrogels have been also employed as green and cost-effective adsorbents for the removal of various contaminants from synthetic aqueous media and wastewater [40,41,42,43].

Herein, we present a unique combination of naturally derived, bio-based materials, namely calcium alginate (CA) and carboxylic acid-functionalized biochar *Luffa cylindrica* (*L. cylindrica*) fibers (ox-LC) within 3D composite hydrogel adsorbents aiming to enhance the adsorption efficiency for U(VI) in comparison to the individual counterparts (i.e., pure CA and pure ox-LC). The produced materials were characterized with respect to their morphology, swelling behavior, mechanical properties, and U(VI) adsorption efficiency as a function of various physicochemical parameters, including uranium concentration, pH, and temperature. The produced biochar-functionalized alginate hydrogel adsorbents demonstrated enhanced adsorption efficacy for U(VI) compared to the pristine ox-LC and hydrogel counterparts. Consequently, the present study paves the way towards the development of cost-effective, naturally derived 3D composites using simple and undemanding synthetic processes, exhibiting enhanced adsorption performance in the removal of radiotoxic metal ions from aquatic environments.

## 2. Experimental

### 2.1. Materials

Alginic acid sodium salt (SA) from brown algae (A2033, Molar Mass = 21,900 g.mol^−1^; mannuronate residues to guluronate residues (M/G) ratio = 2.12 [44]) was purchased from Sigma-Aldrich (Darmstadt, Germany). Calcium chloride anhydrous (97%) was supplied by HiMedia Laboratories (Einhausen, Germany). Both reagents were used in the fabrication of the CA hydrogels without further purification. *L. cylindrica* (LC) fibers were purchased locally, and they were further subjected to a thermochemical treatment as described elsewhere [18]. The oxidized biochar obtained from *L. cylindrica* fibers (ox-LC) was then ground using a pestle and a mortar, and the particle fraction corresponding to the mesh size below 200 μm was used in the fabrication of the CA/ox-LC 3D hydrogel composites. Uranyl nitrate hexahydrate (UO_2_(NO_3_)_2_·6H_2_O, molar mass 105.99 g mol^−1^, 99.9%), which was used in the adsorption studies, and anhydrous sodium carbonate (Na_2_CO_3_, molar mass 502.13 g mol^−1^, 99.9%), which was used for the preparation of the recovery solutions, were supplied by Sigma-Aldrich. In addition, 0.1 M NaOH and 0.1 M HClO_4_ solutions were used for pH adjustments using the corresponding concentrate ampules (Sigma-Aldrich), and the potassium bromide salt (KBr, molar mass 119.00 g mol^−1^, 99.999%) used for the FTIR spectroscopic measurements was also purchased from Sigma-Aldrich.

### 2.2. Adsorbent Fabrication

A pristine CA hydrogel (reference sample) as well as a series of CA/ox-LC composites with variable ox-LC loading were synthesized as described in the following: For the reference sample, SA (250 mg) was placed in a glass vial, and CaCl_2_ (100 mg) was weighted in a separate vial. De-ionized (DI) water (10 mL and 5 mL, respectively) was then added to the two vials, and the resulting mixtures were left to stir at room temperature until the complete dissolution of SA and CaCl_2_. Subsequently, the CaCl_2_ aqueous solution was poured into the glass vial containing the SA aqueous solution, resulting in the formation of the CA hydrogel within a few seconds. The produced hydrogel was left in DI water for 7 days in order to reach its maximum swelling capacity (i.e., equilibrium state).

**Figure 1:**
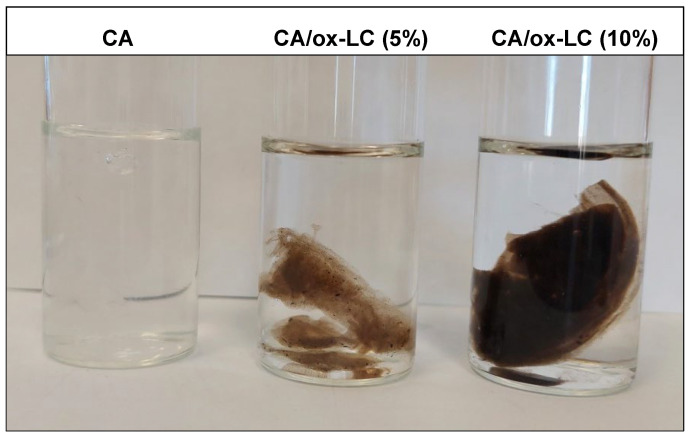
Photographs of the pristine CA and the CA/ox-LC composite hydrogels with 5% and 10% wt. ox-LC content. The black color observed in the case of the ox-LC-containing hydrogels is attributed to the incorporation of the biochar ox-LC fibers.

A similar experimental procedure was followed for the fabrication of a series of CA/ox-LC composite hydrogels with 5% and 10% wt. ox-LC content with respect to the total mass of SA and ox-LC. More precisely, specific quantities of ox-LC (i.e., 14 mg and 28 mg, corresponding to 5% and 10% ox-LC loading percentage) were added to the SA aqueous solution (prepared using 250 mg of SA dissolved in 10 mL of DI water) and left to stir at ambient conditions until a homogeneous SA/ox-LC aqueous dispersion was obtained. This was followed by the addition of the CaCl_2_ aqueous solution (prepared by dissolving 100 mg of CaCl_2_ in 5 mL of DI water) in the resulting SA/ox-LC aqueous dispersion, which led to the instantaneous formation of the SA/ox-LC hydrogel composite. As in the case of the pristine CA hydrogel, the produced composite hydrogels were left in DI water for 7 days to reach their equilibrium swollen state. In Figure 1, characteristic photographs of the pristine CA and the CA/ox-LC composite hydrogels are provided, with the black color observed in the ox-LC-containing hydrogels attributed to the incorporation of ox-LC additives.

### 2.3. Adsorbent Characterization

#### 2.3.1. Morphological Characterization

Scanning Electron microscopy (SEM) (Vega TS5136LS-Tescan, Brno, Czech Republic) was employed to obtain information on the morphological characteristics of the ox-LC and the produced hydrogels (CA and CA/ox-LC). For enabling the visualization of the internal morphology of the hydrogels, lyophilization of the water-swollen hydrogel specimens was performed using a Virtis Genesis 25EL lyophilizer (Surplus Solutions, Woonsocket, RI, USA). Prior to SEM analysis, the ox-LC, CA, and CA/ox-LC samples were Au-sputtered (Au film thickness: 30 nm), using the K575X Turbo Sputter Coater-Emitech sputtering system. Energy Dispersive X-ray Spectroscopy (EDS) (Vega II LSU-Tescan equipped with a Princeton Gamma Tech EDS detector, Brno, Czech Republic) was employed for the detection of Uranium in the U(VI)-loaded CA/ox-LC samples. Multiple EDS analyses (Beam Energy 20 kV, Live Time: 100 s, Take-off angle 45°, Scanning Area: 350^2^ μm^2^) were performed on non-Au coated CA/ox-LC adsorbents that had been previously equilibrated in aqueous solutions with various U(VI) concentrations (1 ∙ 10^−3^, 5 ∙ 10^−4^, 1 ∙ 10^−4^, 5 ∙ 10^−5^, and 5 ∙ 10^−6^ M) and the corresponding average Uranium mass fractions (U(wt%)) were calculated. Elemental analyses were based on the standardless (ZAF matrix correction) quantification protocol.

#### 2.3.2. Mechanical Properties

Compression tests were performed on the water-swollen CA (reference sample) and CA/ox-LC composite (10% wt. ox-LC content) (Instron 5944, Norwood, MA, USA). Specifically, two specimens from each sample (dimensions: 4.24 mm × 5.6 mm × 6.26 mm, length × width × thickness, respectively) were analyzed, and average values along with standard deviations were calculated. The specimens were compressed to 30% strain with a strain rate of 0.1 mm·min^−1^. The Young’s modulus was calculated from the slope of the linear part of the stress–strain curves.

#### 2.3.3. Swelling Behavior

After reaching the equilibrium state, the water-swollen hydrogels were cut into small pieces, and their water-swollen mass was determined gravimetrically. Subsequently, the water-swollen hydrogel pieces were placed in a vacuum oven at 40 °C for 7–9 h. The dry hydrogel mass was then determined, and the swelling ratio was calculated as the ratio of the swollen mass (W_wet_) divided by the dry mass (W_dry_).

### 2.4. Adsorption Studies

Adsorption studies were conducted to evaluate the adsorption efficiency of the CA and CA/ox-LC hydrogel composites in the removal of U(VI) ions from aqueous media. Batch-type experiments were performed in order to investigate the effect of different physicochemical parameters, including the initial metal ion concentration, as well as the effect of solution pH and temperature, on the adsorption capacity of the hydrogels. For the investigation of every aforementioned parameter, a certain amount of adsorbent was used (0.01 g) under normal atmospheric conditions, while the rest of the parameters were kept constant. Adsorption studies were conducted using ultraviolet-visible spectrophotometry (UV-Vis—UV 2401 Shimadzu, Shimadzu, Columbia, MD, USA), based on the Arsenazo III method [45]. In all cases, the experimental data provided are average values obtained from three measurements. Their low uncertainty (<10%) can be ascribed to the error associated with spectrophotometry, which was carried out in the presence of arsenazo-III. In addition, after the completion of the U(VI) adsorption process, the material exhibiting the highest adsorption capacity was further analyzed by FTIR spectroscopy (FTIR spectrometer 8900, Shimadzu, Shimadzu, Columbia, MD, USA). Finally, adsorption/desorption cycles were performed using carbonate solutions, aiming to investigate the potential recovery of the adsorbed U(VI) and reuse of the hydrogel adsorbents.

#### 2.4.1. Effect of Initial Metal Ion Concentration

To investigate the effect of the initial metal ion concentration, a specific amount of the hydrogel-based adsorbent (10 mg) was placed in aqueous solutions (30 mL) prepared in DI water, containing various U(VI) concentrations (1 ∙ 10^−3^, 5 ∙ 10^−4^, 1 ∙ 10^−4^, 5 ∙ 10^−5^ and 5 ∙ 10^−6^ M). In all cases, the pH value was adjusted to 3 [36]. Samples were left to stir for 24 h under normal atmospheric conditions. Following that, a specific amount (0.5 mL) was taken out of the supernatant solution, and an additional amount of arsenazo-III (2.5 mL) was added, reaching a final volume of 3 mL. UV-vis analysis was then employed to determine the quantity of the adsorbed U(VI), i.e., the adsorption capacity, q_e_ (mg·g^−1^). Equations (1) and (2) were used in the calculation of *q_e_* and the relative removal efficiency (% *q_e_*) [19]:

(1) $$q_{e}=\frac{{\left[U\left(VI\right)\right]}_{0}-{\left[U\left(VI\right)\right]}_{aq}}{m}\cdot V$$

(2) $$q_{e}\%=\frac{{\left[U\left(VI\right)\right]}_{0}-{\left[U\left(VI\right)\right]}_{aq}}{{\left[U\left(VI\right)\right]}_{0}}\cdot 100$$

where U(VI)_0_ is the initial uranium concentration in the system and U(VI)_aq_ is the uranium concentration in solution after the adsorption process has reached equilibrium.

#### 2.4.2. pH Effect

To investigate the effect of pH, aqueous U(VI) solutions of known total metal ion concentration ([U(VI)]_0_ = 1 × 10^−4^ M) were prepared, in which pH was varied between 2.1 and 7.3. The pH was adjusted by the addition of NaOH or HClO_4_ (0.1 M to 1 M). Measurements were taken using a glass electrode pH-meter, (Hanna Instruments pH 211) after 24 h incubation, and the analytical metal ion concentration in solution was determined spectrophotometrically (UV 2401 PC Shimadzu) using arsenazo-III [45].

#### 2.4.3. Temperature Effect

Temperature is another physicochemical parameter that affects the system, and throughout this study, thermodynamic parameters such as change in enthalpy (Δ*H*), and entropy (Δ*S*) were determined, using Equation (3), where *K_d_* is the distribution coefficient (Equation (4)), *R* is the universal gas constant, and *T* is the temperature. The experimental procedure followed, involved the use of U(VI) aqueous solutions (30 mL, metal ion concentration: 1 ∙ 10^−4^ M), prepared pH = 3, in which the hydrogel adsorbent (10 mg) was immersed and its adsorption efficiency was determined at 20, 27, 36 and 45 °C. The particular temperatures were controlled and maintained using an orbital shaker incubator (Gallenkamp, Cambridge, UK).

(3) $$lnK_{d}=\frac{-\Delta H{}^{\circ}}{R\times T}+\frac{\Delta S{}^{\circ}}{R}$$

(4) $$K_{d}=\frac{q_{e}}{C_{e}}\;(\frac{L}{kg})$$

#### 2.4.4. FTIR Analysis

The evaluation of the surface-active moieties of the adsorbent and their speciation after U(VI) adsorption was carried out by FTIR spectroscopic measurements using an FTIR spectrometer 8900 (Shimadzu). Aliquots of the hydrogel composite with the highest adsorption capacity were dried overnight in a vacuum oven at 80 °C, and the following FTIR probes were prepared in the form of translucent KBr disks, including finely ground composite encapsulated at a 10:1 mass ratio.

#### 2.4.5. Uranium Recovery

U(VI) recovery investigations were performed by batch-type experiments using carbonate solutions (0.1 M Na_2_CO_3_, pH 11). Strong acid and EDTA solutions have not been used to avoid calcium dissolution and extensive alteration of the adsorbent. After completing the adsorption experiments, the solid phase (0.01 g) was separated from the suspension, and 30 mL of the carbonate solution was added. Subsequently, the new suspension was agitated overnight in an orbital shaker (100 rpm, ambient conditions), the uranium concentration in solution was determined by UV-Vis spectrophotometry, and the desorption efficiency (% Desorption) was calculated using the following Equation:

(5) $$\%\;Desorption=\frac{[U(VI){]}_{des}}{[U(VI){]}_{ads}}\times 100$$

where [U(VI)]*_des_* is the concentration of uranium in the recovery solution and [U(VI)]*_ads_* the uranium concentration that corresponds to the uranium amount adsorbed and is calculated from the uranium concentration prior to adsorption minus the uranium concentration after adsorption. It has to be noted that both adsorption and desorption solutions had the same volume (30 mL).

## 3. Results and Discussion

### 3.1. Adsorbent Fabrication and Characterization

The synthesis of CA hydrogels is based on a physical crosslinking process involving the interconnection of the carboxyl groups that are incorporated within the SA linear chains with Ca^2+^, acting as crosslinking agents. Consequently, the 3D hydrogels are instantaneously formed upon mixing the SA and CaCl_2_ aqueous solutions, due to the development of strong electrostatic attractive forces between the hydroxyl and (mainly) the deprotonated carboxylic moieties and the Ca^2+^ ions [46]. Besides the presence of carboxyl functionalities within the SA linear chains, in the case of the ox-LC-functionalized materials, the surface modification of carbonized LC fibers results in the generation of carboxyl groups, thus providing additional binding sites during the crosslinking process. This is further dictated by the decrease in the swelling ratio of the produced hydrogels upon increasing the content of ox-LC, as seen in Table 1.

**Figure 2:**
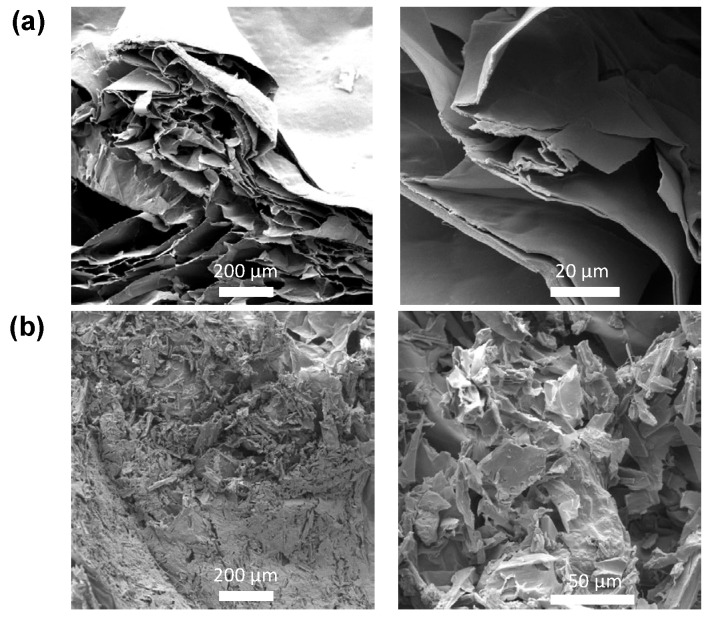
SEM images of pristine CA hydrogel (reference sample) (**a**) and of the CA/ox-LC composite hydrogel (10 % wt. ox-LC) (**b**).

Scanning Electron Microscopy (SEM) further confirmed the aforementioned, since, as seen from the SEM images provided in Figure 2a,b, the internal morphology of the CA/ox-LC hydrogel composite (with 10% wt. ox-LC content) exhibited a more compact (less porous) structure, while triturated fibers could also be visualized all over the hydrogel’s surface.

**Figure 3:**
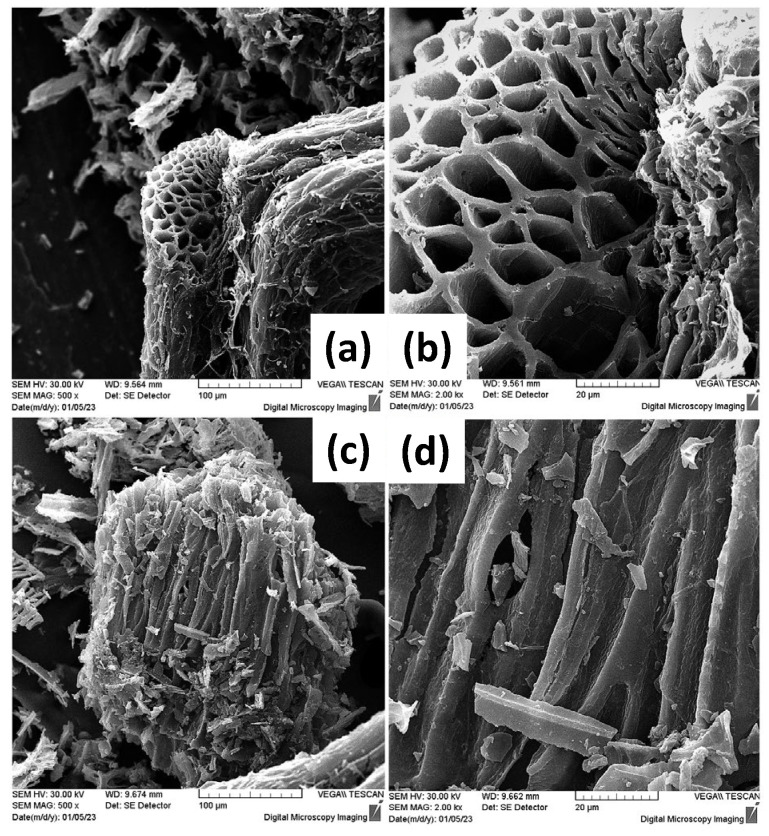
SEM images of the ground and Au-coated (~30 nm) ox-LC additive samples presenting distinctive tubular microchannel structures (**a**–**d**).

SEM was also employed in the visualization of the morphology of the ox-LC additives after grinding. As seen in Figure 3, ox-LC consists of microchannels, whereas the obtained morphology resembles that of the non-carbonized material [47]. The latter demonstrates that the material’s morphology is not significantly altered upon carbonization. Even after grinding—that was applied to achieve higher homogeneity with respect to the distribution of ox-LC within CA hydrogel—it was observed that the additives’ morphology was retained, in line with previous observations [48].

**Figure 4:**
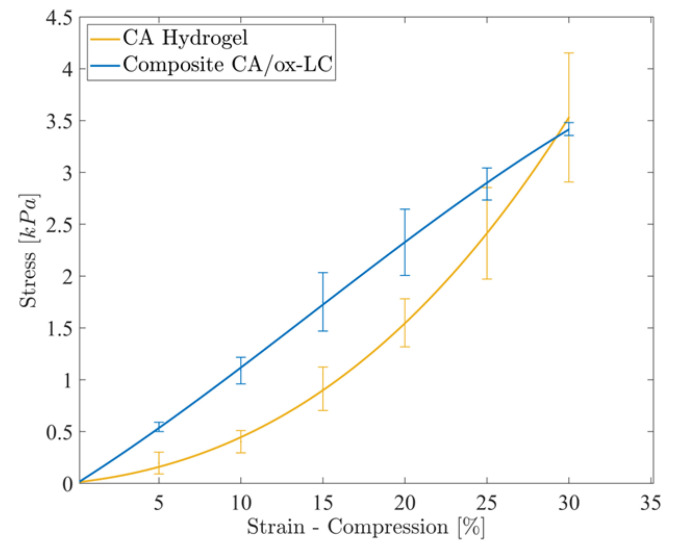
Stress-strain experimental curves corresponding to the CA and CA/ox-LC (10% wt.) hydrogel adsorbents, recorded under unconfined compression.

The mechanical behavior of the ox-LC-free and ox-LC-loaded CA hydrogels was investigated by performing compression tests on the water-swollen specimens. The stress-strain curves corresponding to CA and CA/ox-LC (10% wt.) hydrogel adsorbents are provided in Figure 4. As seen, the incorporation of ox-LC within the CA hydrogels results in increased stiffness, as the stress recorded in the case of the composite hydrogel is higher in comparison to the pristine CA (reference sample) for the same strain, resulting in a higher value of Young’s modulus, i.e., 110 ± 20 kPa compared to 30 ± 5 kPa corresponding to the pure CA hydrogel. These results are in line with the swelling behavior, since upon increasing the ox-LC content, the swelling ratio decreases, resulting in a further increase in the materials’ stiffness. This result could be attributed to the development of interactions between the hydrogel matrix and the embedded ox-LC, which caused a reduction in the deformability of the resulting composites compared to the pure hydrogel. Such interactions were promoted via surface functionalization/oxidation of the embedded biochar fibers obtained from *L. cylindrica*, which assisted the homogeneous distribution of the carbon additives within the hydrogel, resulting in increased stiffness. As a consequence, a transition from a nonlinear (elastomeric) behavior to a linear elastic behavior could be observed in the presence of ox-LC.

In most environmental applications where hydrogels are involved as adsorbents, such as water remediation, the long-term preservation of the materials’ structural integrity, stability, and robustness under continuous mechanical stress is of paramount importance. Consequently, the fact that the presence of c-LC within the CA hydrogels enhances the mechanical properties of these materials is considered advantageous for their potential use as adsorbents in wastewater treatment.

### 3.2. Adsorption Studies

The pristine CA as well as the composite CA/ox-LC hydrogel analogues with variable ox-LC content were evaluated as adsorbents for the removal of U(VI) from synthetic aqueous media. The adsorption efficiency was evaluated as a function of pH, temperature, and initial metal ion (U(VI)) concentration.

#### 3.2.1. Effect of pH

**Figure 5:**
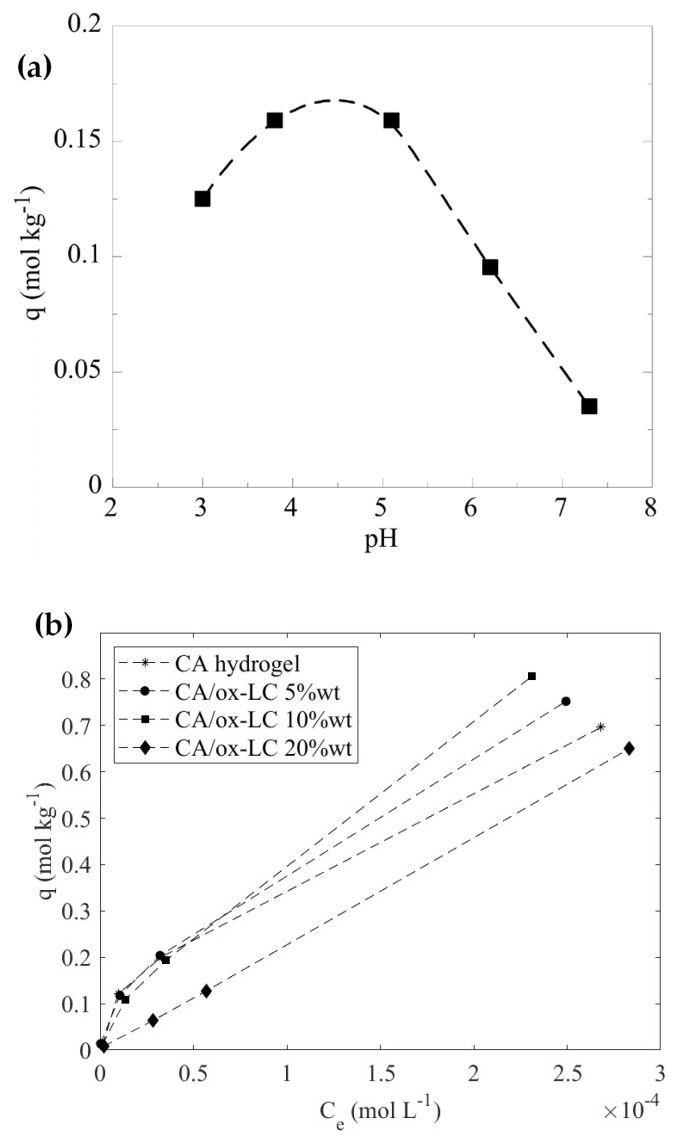
Effect of pH on the U(VI) adsorption on CA/ox-LC composite hydrogel (10% wt. ox-LC) (10 mg adsorbent in 10 mL solution, [U(VI)]_tot_ = 1 · 10^−4^ M, T = 23  ±  2  °C) (**a**) and adsorption isotherms corresponding to U(VI) adsorption by CA pristine and CA/ox-LC composite hydrogels containing 5%, 10% and 20% wt. ox-LC from aqueous media. Solution pH = 3 (**b**).

The solution pH is a main parameter affecting the adsorption capacity because it determines both the dissociation of the surface-active groups (e.g., carboxylic and hydroxyl groups) and hence the surface charge of the composite adsorbent and the speciation of U(VI) in solution, which is associated with the stability of the U(VI) species in solution. The effect of pH on the adsorption capacity of U(VI) by the studied composite material is shown in Figure 5.

According to Figure 5a, the adsorption capacity increases gradually with pH up to pH 5, because the acidic surface-active groups of the composite material (e.g., carboxylic acids) dissociate increasingly with pH, resulting in a steady rise of the negative charge on the composite’s surface, which progressively attracts positively charged U(VI) species such as UO_2_^2+^ and UO_2_OH^+^) which dominate below pH 5. Above pH 5, the adsorption capacity declines with pH because of the negatively charged U(VI) carbonate complexes, which become predominant in solution and are electrostatically repelled by the similarly charged surface, disfavoring adsorption and stabilizing U(VI)-carbonate complexes in solution [49].

#### 3.2.2. Effect of Initial Metal Ion Concentration

The effect of the initial U(VI) concentration was investigated using a pristine CA hydrogel (reference sample) and two different CA/ox-LC composites with variable ox-LC loading (e.g., 5% and 10%). According to Figure 5b, which shows the corresponding adsorption data obtained, it is obvious that the composite materials present higher adsorption capacity.

The adsorption capacity depends on the biochar content in the composite, with the 10% wt. content possessing the highest adsorption capacity with a value equal to 1.7 mol·kg^−1^ (404.6 mg · g^−1^) at pH 3. This value, which has been evaluated by fitting the experimental data using the Langmuir adsorption model, is significantly higher than the corresponding values for the pristine CA (335.6 mg · g^−1^) and the oxidized LC fibers (92.0 mg · g^−1^) [12], indicating the enhancement of the adsorption capacity of the composite compared to the precursor materials. As seen in Figure 5, by further increasing the ox-LC content, reaching 20 wt%, the adsorption capacity is significantly reduced. This phenomenon might be attributed to the occurrence of ox-LC aggregation phenomena during gelation, which in turn reduces the active surface area of the embedded additives and consequently the U(VI) adsorption efficiency.

In addition, the composite presents remarkably higher adsorption capacity value than those reported in the literature for related systems under similar conditions. Table 2 summarizes the adsorption capacity data recorded for related U(VI) adsorbents, including hydrogel, biochar, hydrogel-biochar, and other hydrogel-based composite adsorbents. A comparison of the q_max_ determined in the present study using the Langmuir adsorption model with corresponding literature values clearly shows that in most cases, the CA/ox-LC hydrogel composite exhibits higher adsorption capacity compared to similar adsorbents.

Furthermore, the experimental data corresponding to the pristine CA hydrogel (reference sample) and the 10% wt CA/ox-LC composite were fitted using the Langmuir and Freundlich isotherm models.

**Figure 6:**
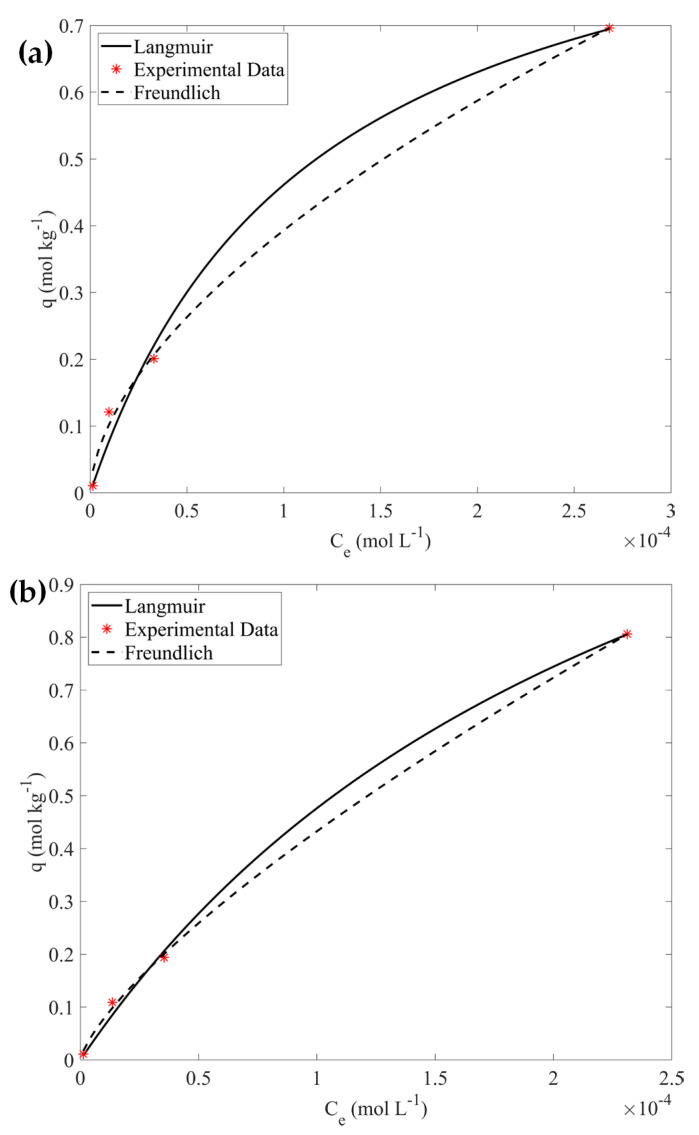
Adsorption data for CA hydrogel (**a**) and composite CA/ox-LC (**b**) fitted using the Langmuir and the Freundlich isotherm models.

According to the data shown in Figure 6 and the associated data summarized in Table 3, the data for the pristine hydrogel and the composite material are well-fitted by both models and the q_max_ values evaluated are 0.99 mol kg^−1^ and 1.70 mol kg^−1^, respectively.

Due to the highest adsorption capacity of CA/ox-LC (10% wt.) hydrogel composite adsorbent, this material was chosen for performing further studies in order to investigate the effect of temperature on the material’s adsorption efficiency.

#### 3.2.3. Morphological Characterization of the U(VI)-Loaded CA/ox-LC Adsorbents

**Figure 7:**
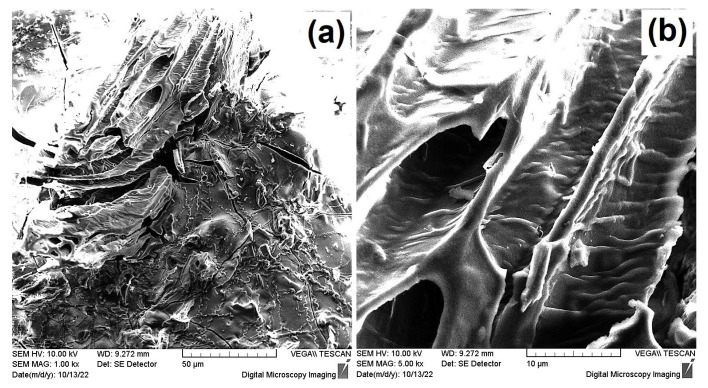
SEM images of non-Au coated U(VI)-loaded CA/ox-LC adsorbent presenting morphological similarities to U(VI)-free CA/ox-LC samples (**a**); A detailed view of the CA hydrogel-covered LC microchannel is also depicted (**b**).

Evaluation of the morphology of the U(VI)-loaded CA/ox-LC adsorbents that had been previously equilibrated with an aqueous U(VI) solution ([U(VI)] = 1 × 10^−3^ mol L^−1^) was enabled through SEM. As can be seen in Figure 7, the U(VI)-loaded adsorbent presents a similar morphology to that of the pristine (U(VI)-free) CA/ox-LC samples (Figure 2b), with a compact CA hydrogel surface covering the LC microchannels.

#### 3.2.4. Elemental Analysis of the U(VI)-Loaded CA/ox-LC Adsorbents

Elemental characterization of non-Au-coated U(VI)-loaded CA/ox-LC adsorbents, that had been previously equilibrated with aqueous U(VI) solutions of varying Uranium concentration (1 ∙ 10^−3^, 5 ∙ 10^−4^, 1 ∙ 10^−4^, 5 ∙ 10^−5^, and 5 ∙ 10^−6^ M), was also enabled through SEM-EDS X-ray microanalysis. Multiple area scans were performed on these samples, and the corresponding EDS spectra were extracted. Besides Uranium, which was analyzed based on the Ma1 characteristic line, all other elements shown in the EDS spectra (Figure S1a–e, Supplementary Materials, SM) were analyzed based on their Ka1 lines.

As can be seen from the indicative EDS spectra in Figure S1a–e (SM), all samples present major peaks associated with C and O and minor peaks associated with Ca, Cl, and U. Both C and O can be related to the organic content of the U(VI)-loaded CA/ox-LC adsorbents and to the carbon tape used for mounting the samples on the Al stabs. On the other hand, the presence of Ca and Cl can be related to the CaCl_2_ reagent used as a crosslinking agent in the fabrication of the CA hydrogels. In some cases, small amounts of Al, Si, and Mg were detected, the origin of which is believed to be contamination. Minor and trace amounts of Na, close to and below the detection limit of the EDS measurement, were also observed in all samples.

**Figure 8:**
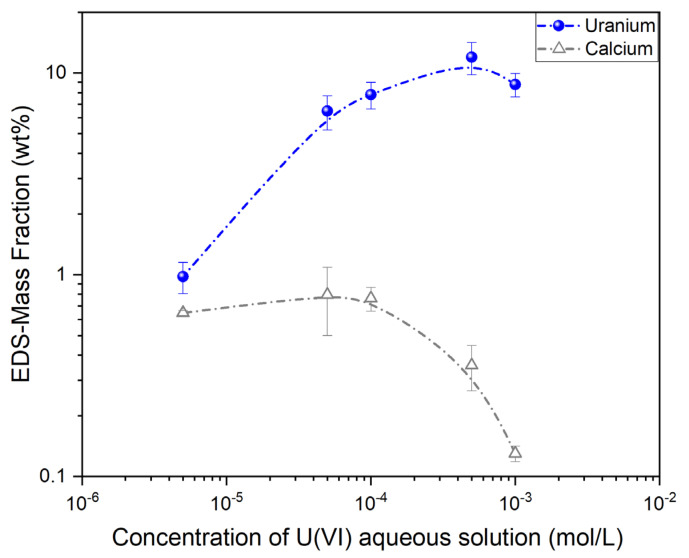
EDS-derived data of the average U (wt%) in the U(VI)-loaded adsorbents as a function of the U(VI) concentration in the aqueous solutions. Corresponding changes in the Ca (wt%) are also shown.

The detection of Uranium on the surface of all samples can be associated with adsorption that took place after equilibration of the CA/ox-LC composites with aqueous U(VI) solutions of varying Uranium concentration. As can be seen in Figure 8, the mass fraction of Uranium presents an increasing and saturating trend with increasing U(VI) concentration in the aqueous solution. Interestingly, the Ca mass fraction appears to decrease with an increasing U mass fraction.

It must be noted that unpredictable errors can be introduced in these measurements due to the highly textured samples [61]. Thus, inferences regarding an interdependence between the mass fractions of U and Ca based on these semi-quantitative analyses cannot be safely made. Nevertheless, changes in the average mass fractions (wt%) of U and Ca as a function of the U(VI) concentration in the aqueous solution are illustrated in Figure 8. Error bars correspond to the mass fraction average deviation.

#### 3.2.5. Temperature Effect

**Figure 9:**
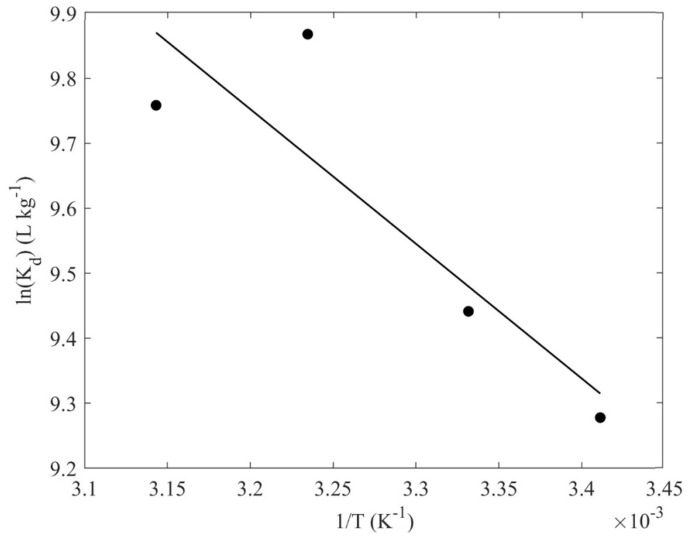
lnK_d_ as a function of (1/T) for the adsorption of U(VI) by the CA/ox-LC composite hydrogel (10% wt. ox-LC) (10 mg adsorbent in 10 mL solution, [U(VI)]_tot_ = 1 × 10^−4^ mol L^−1^, 24 h of contact time, at pH 3).

In Figure 9, the data obtained from the effect of temperature on the sorption capacity are summarized in the form of a lnK_d_-1/T plot. The adsorption of uranium by the composite material is an endothermic and entropy-driven process, implying increased randomness at the solid–liquid interface during adsorption, which is associated with the release of several water molecules upon binding between the uranyl cation and the carboxylic or hydroxy surface groups. Evaluation of the respective data results in ΔH^o^ and ΔS^o^ equal to 6.5 kJ mol^−1^ and 93.4 J mol^−1^, respectively. These values are similar to corresponding values obtained from investigations related to U(VI) adsorption by chitosan-blended nanofibers [21], and U(VI) adsorbed by oxidized biochar obtained from natural fibers [17,62].

#### 3.2.6. FTIR Analysis and Adsorbent Recovery

**Figure 10:**
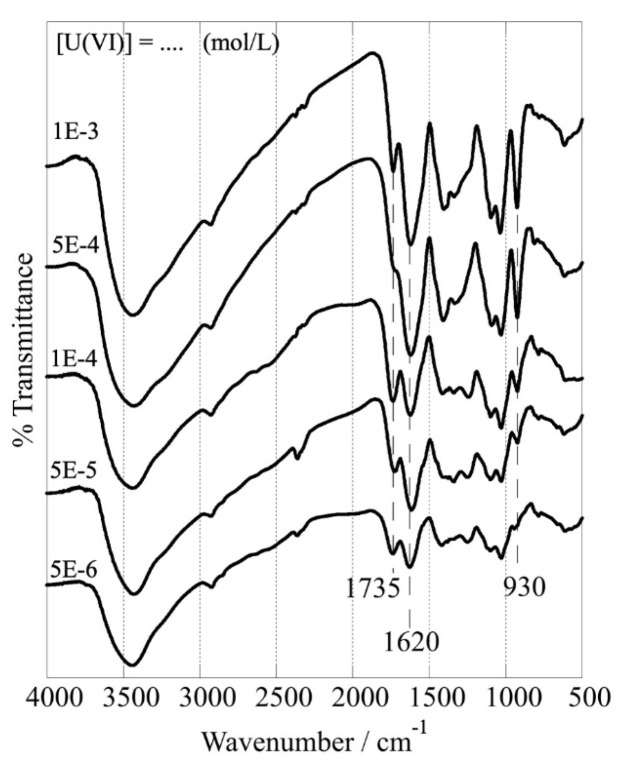
FTIR spectra of CA/ox-LC composite hydrogel (10% wt. ox-LC) after U(VI) adsorption at different initial U(VI) concentrations (10 mg adsorbent in 10 mL solution), [U(VI)]tot = [5 × 10^−6^ – 10^−3^] mol L^−1^, temperature at 298 K, 24 h of contact time, at pH 3).

Upon U(VI) adsorption, the FTIR spectra of the CA/ox-LC (10% wt.) composite hydrogel adsorbent change dramatically (Figure 10), particularly the peaks corresponding to carbonyl/carboxylic moieties, indicating the formation of inner-sphere complexes between U(VI) and the respective surface-active groups. According to the spectra shown in Figure 10, by increasing the amount of U(VI) adsorbed, the peaks corresponding to carbonyl stretching (1735 cm^−1^), carboxylic antisymmetric stretching (1620 cm^−1^), and carboxylic bending vibrations (peaks between 1500 and 1000 cm^−1^) change basically in their relative intensity. Moreover, the intensity of the peak at 930 cm^−1^, which is attributed to the stretching vibration of the O=U=O moiety [18,19], increases gradually because of the increasing amount of U(VI) adsorbed.

Data obtained from preliminary experiments related to material recycling and uranium recovery using acidic or EDTA solutions have shown that recycling of the material in its present form is limited due to material swelling and deterioration of the composite. On the other hand, uranium recovery using carbonate solutions (0.1 M Na_2_CO_3_) was relatively low (~10%), indicating strong binding of U(VI) by the surface-active groups and its incorporation in the composite matrix.

## 4. Conclusions

In summary, a facile, eco-friendly, and cost-effective synthetic process was followed to prepare composite hydrogel adsorbents based on calcium alginate (CA) hydrogels and oxidized biochar obtained from *L. cylindrica* fibers (ox-LC). The resulting naturally−derived 3D composites were further evaluated as adsorbents for the removal of U(VI) from synthetic aqueous media. The best adsorption performance was obtained at pH 3, in the case of the CA/ox-LC composite hydrogel containing 10% wt. ox-LC, which demonstrated enhanced adsorption capacity (1.7 mol · kg^−1^, corresponding to 404.6 g · kg^−1^) compared to either pure CA hydrogels (0.99 mol · kg^−1^, corresponding to 335.6 g · kg^−1^) or pristine ox-LC fibers (0.4 mol · kg^−1^, corresponding to 92 g · kg^−1^ [18]. Thermodynamic parameters indicated an endothermic and entropy-driven adsorption process. Despite its remarkable adsorption capacity, the composite cannot be properly recycled and reused due to its extensive deterioration during the U(VI) recovery. Hence, our future efforts will be focused on the preparation of more stable composite forms.

## Figures and Tables

**Table 1:** Swelling ratios (recorded in DI water) and corresponding standard deviations (SD) of the pristine CA (0% wt. ox-LC) and CA/ox-LC composite hydrogels with 5 and 10% wt. ox-LC content.

Adsorbent	Swelling Ratio and SD
CA	41 ± 0.6
CA/ox-LC (5% wt)	35 ± 1.5
CA/ox-LC (10% wt)	30 ± 1.0

**Table 2:** Adsorption capacity data of various U(VI) adsorbents.

Adsorbent	pH	q_max_ (mg·g^−1^)	Literature
**Hydrogel-based adsorbents**			
Calcium Alginate beads	7.0	237.2	[50]
Semi-interpenetrating Alginate-based microspheres	8.1	186.6	[51]
Cellulose Hydrogel	3.0	148.0	[52]
Poly(acrylic acid) hydrogel	4.0	445.1	[53]
Guar gum/acrylamide/orange peel biochar hydrogel	5.5	263.2	[54]
Graphene oxide-doped double network hydrogels	6.0	625.0	[55]
ZnO-modified biochar-based hydrogels	5.0	239.2	[56]
Amidoxime- functionalized magnetoactive microspheres	4.5	200.5	[57]
**Biochar-based adsorbents**			
Activated Biochar fibers	7.0	210	[19]
Surface-oxidized Biochar obtained from LC fibers	3.0	92.0	[18]
Oxidized biochar obtained from palm tree fibers	6.0	112.0	[17]
Carboxyl and amidoxime-modified LC fibers	5.0	399.1	[58]
Manure-derived biocarbon doped with TiO_2_ and SiO_2_	4.5	675.1	[59]
Magnetic sulfhydryl-functionalized biomass carbon	7.0	273.0	[60]
CA hydrogel	3.0	335.6	**This study**
CA/ox-LC composite hydrogel	3.0	404.6	**This study**

**Table 3:** Adsorption constants determined by applying the Langmuir and Freundlich isotherm models to the experimental adsorption data.

Freundlich			
**Sample**	**K_F_ [L/mg]**	**n**	**R^2^**
10% wt LC fibers	3.99 · 10^2^	1.35	0.999
CA hydrogel	8.13 · 10^1^	1.72	0.993
**Langmuir**			
**Sample**	**K_L_ [L/mg]**	**q_max_ [mol kg^−1^]**	**R^2^**
10% wt LC fibers	3.89 · 10^3^	1.70	0.999
CA hydrogel	8.68 · 10^3^	0.995	0.993

## Data Availability

The data presented in this study are available upon request from the corresponding author.

## Supplementary Materials

The following supporting information can be downloaded at: https://www.mdpi.com/article/10.3390/ma16196577/s1, Figure S1: Representative EDX spectra of CA/ox-LC samples that have been previously equilibrated with aqueous solutions of U(VI) at varying concentrations: (a) 5 × 10^−6^ M, (b) 5 × 10^−5^ M, (c) 1 × 10^−4^ M, (d) 5 × 10^−4^ M and (e) 1 × 10^−3^ M.
