# Supplementary material for: 3D Composite U(VI) Adsorbents Based on Alginate Hydrogels and Oxidized Biochar Obtained from Luffa cylindrica
Source: Materials (Basel). 2023 Oct 6;16(19):6577. doi: 10.3390/ma16196577 (PMC10574392; doi:10.3390/ma16196577)
Supplement: Supplementary file 1 [file materials-16-06577-s001.zip › materials-2623267-supplementary.pdf]

materials

3D composite U(VI) adsorbents based on alginate hydrogels and oxidized biochar obtained from *Luffa Cylindrica*

Andreas Ayiotis[1]; Efthalia Georgiou[2]; Panagiotis S. Ioannou[1]; Ioannis Pashalidis[2] and Theodora Krasia-Christoforou[1*]

[1] Department of Mechanical and Manufacturing Engineering, University of Cyprus, 1 Panepistimiou Avenue, 2109, Aglantzia, Nicosia, P. O. Box 20537, 1678 Nicosia, Cyprus; ayiotiss@gmail.com (A.A.); ioannou.s.panagiotis@ucy.ac.cy (P.S.I.)
[2] Department of Chemistry, University of Cyprus, 1 Panepistimiou Avenue, 2109, Aglantzia, Nicosia, P. O. Box 20537, 1678 Nicosia, Cyprus; georgiou.efthalia@ucy.ac.cy (E.G.); paschalidis.ioannis@ucy.ac.cy (I.P.)
* Correspondence: krasia.theodora@ucy.ac.cy

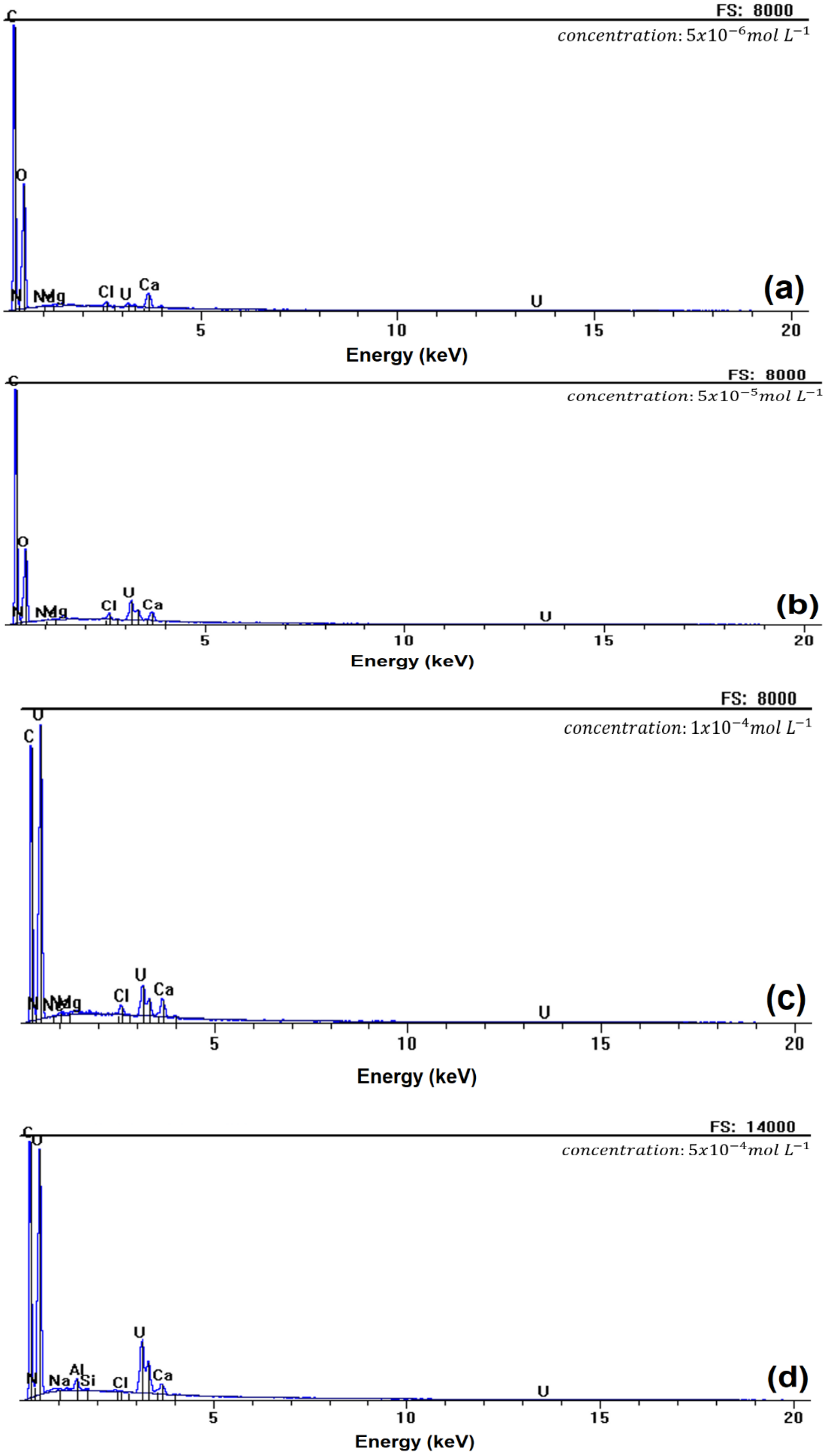

FS: 8000
concentration: $5x10^{-6} mol\ L^{-1}$
C
O
Cl
U
Ca
U
(a)
5
10
15
20
Energy (keV)
FS: 8000
concentration: $5x10^{-5} mol\ L^{-1}$
C
O
Cl
U
Ca
U
(b)
5
10
15
20
Energy (keV)
FS: 8000
concentration: $1x10^{-4} mol\ L^{-1}$
U
C
Cl
U
Ca
U
(c)
5
10
15
20
Energy (keV)
FS: 14000
concentration: $5x10^{-4} mol\ L^{-1}$
C
U
U
Na
Al
Si
Cl
Ca
U
(d)
5
10
15
20
Energy (keV)

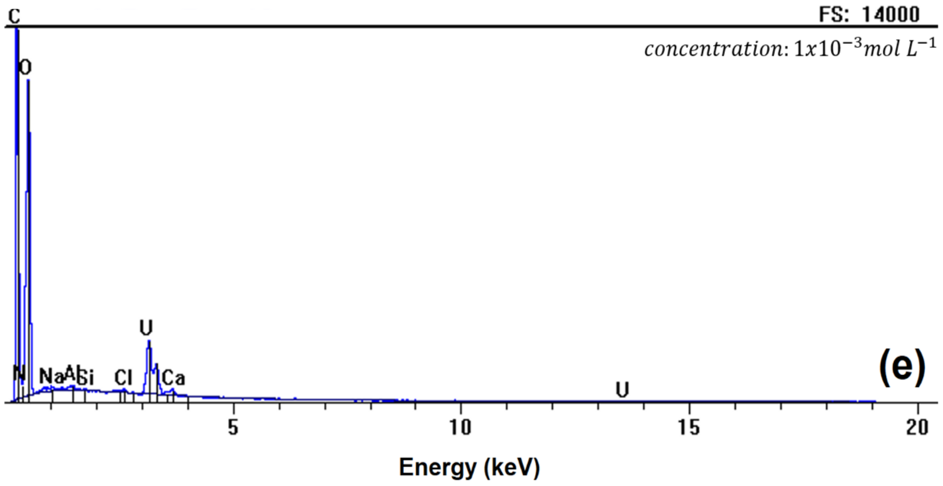

Figure S1. Representative EDS spectra of CA/ox-LC samples that have been previously equilibrated with aqueous solutions of U(VI) at varying concentrations: (a) $5x10^{-6}$ M, (b) $5x10^{-5}$ M, (c) $1x10^{-4}$ M, (d) $5x10^{-4}$ M and (e) $1x10^{-3}$ M.
